# Similar Impacts of the Interaural Delay and Interaural Correlation on Binaural Gap Detection

**DOI:** 10.1371/journal.pone.0126342

**Published:** 2015-06-30

**Authors:** Lingzhi Kong, Zilong Xie, Lingxi Lu, Tianshu Qu, Xihong Wu, Jun Yan, Liang Li

**Affiliations:** 1 Department of Psychology, PKU-IDG/McGovern Institute for Brain Research, Peking University, Beijing Institute for Brain Disorders, Beijing, PR China; 2 Speech and Hearing Research Center, Key Laboratory on Machine Perception (Ministry of Education), Peking University, Beijing, PR China; 3 Hotchkiss Brain Institute, Department of Physiology and Pharmacology, Cumming School of Medicine, University of Calgary, Calgary, AB, Canada; Harvard Medical School/Massachusetts General Hospital, UNITED STATES

## Abstract

The subjective representation of the sounds delivered to the two ears of a human listener is closely associated with the interaural delay and correlation of these two-ear sounds. When the two-ear sounds, e.g., arbitrary noises, arrive simultaneously, the single auditory image of the binaurally identical noises becomes increasingly diffuse, and eventually separates into two auditory images as the interaural correlation decreases. When the interaural delay increases from zero to several milliseconds, the auditory image of the binaurally identical noises also changes from a single image to two distinct images. However, measuring the effect of these two factors on an identical group of participants has not been investigated. This study examined the impacts of interaural correlation and delay on detecting a binaurally uncorrelated fragment (interaural correlation = 0) embedded in the binaurally correlated noises (i.e., binaural gap or break in interaural correlation). We found that the minimum duration of the binaural gap for its detection (i.e., duration threshold) increased exponentially as the interaural delay between the binaurally identical noises increased linearly from 0 to 8 ms. When no interaural delay was introduced, the duration threshold also increased exponentially as the interaural correlation of the binaurally correlated noises decreased linearly from 1 to 0.4. A linear relationship between the effect of interaural delay and that of interaural correlation was described for listeners participating in this study: a 1 ms increase in interaural delay appeared to correspond to a 0.07 decrease in interaural correlation specific to raising the duration threshold. Our results imply that a tradeoff may exist between the impacts of interaural correlation and interaural delay on the subjective representation of sounds delivered to two human ears.

## Introduction

Interaural correlation measures the similarity of the sounds at the two ears, defined as the maximum cross-correlation coefficient of these two sounds. Interaural correlation processing is critical to both localization of auditory objects [1,2] and detection of a target auditory object in a noisy environment [3–7]. The auditory image of the simultaneously-arrived binaural sounds changes dramatically from a single image located at the center area of the head into two separated images at each ear when the interaural correlation decreases from 1 to 0 [8,9]. An understanding of the processing of the interaural correlation however, is incomplete without considering the impact of the interaural delay. When the interaural delay increases from zero to several milliseconds, the single auditory image becomes increasingly diffuse, and eventually indistinguishable from the sound image of the binaurally independent noises [10,11]. In addition, the modulation of auditory neural response by interaural delay is diminished as the interaural correlation decreases [12–15]. The relationship of the effects of these two factors, interaural delay and interaural correlation, has not yet been investigated on an identical group of participants.

Interaural correlation processing can be investigated by measuring the sensitivity to a binaurally uncorrelated noise fragment embedded in the binaurally correlated noises, i.e., a change of interaural correlation from 1 to 0, then back to 1 (i.e., binaural gap or break in interaural correlation). The binaural gap does not alter the energy and spectrum of the binaural noises, but modifies the auditory image, i.e., the perceptual compactness/diffuseness, number, placement, loudness, and the pitch of the noise object determined by interaural correlation [1,16–19]. The binaural gap is detected when the contrast in the perceived interaural correlation between the binaural gap and the markers (the noise sections flanking the binaural gap) is sufficiently large. Measuring the minimum duration of a detectable binaural gap (i.e., duration threshold) has been extensively used to investigate interaural correlation processing (higher duration threshold means lower sensitivity, and vice versa) [20–24].

Human listeners are highly sensitive to the binaural gap when the binaural sounds arrive simultaneously [20,21]. When an interaural delay is introduced, the binaural gap is still detectable [22–27], and the duration threshold increases monotonically as the interaural delay increases to several milliseconds [22–24]. Whether the duration threshold for detecting the binaural gap is also affected by the interaural correlation of the marker (marker correlation) has not been reported in the literature.

This study investigates interaural correlation processing by examining the effects of interaural delay and interaural correlation on detecting the binaural gap. We found that the duration threshold increased exponentially under two specific conditions: when the interaural delay increased from 0 to 8 ms or when the interaural correlation of the marker decreased from 1 to 0.4. A linear relationship between the effects of interaural delay and correlation was described.

## Materials and Methods

### Participants

Six university students (4 females and 2 males, 22–28 years old, mean age = 25 years) with normal hearing participated in this study. Their pure-tone thresholds were no higher than 20 dB HL between 0.125 and 8 kHz [28] and the threshold difference between the two ears at each frequency was less than 15 dB HL. They gave their written informed consent to participate in this study and were paid a modest reward for their participation. All the experiments in this study involving human participants were approved by the Committee for Protecting Human and Animal Subjects in the Department of Psychology at Peking University.

### Apparatus and Stimuli

#### Experiment 1

The participant was seated in a chair at the center of a sound-attenuated chamber (EMI Shielded Audiometric Examination Acoustic Suite). Gaussian wideband noises (0–10 kHz), 1000 ms in duration, including 30-ms rise and fall times, were synthesized using the “randn()” function in MATLAB (the MathWorks Inc., Natick, MA, USA), featuring a 48 kHz sampling rate and 16-bit amplitude quantization. Stimuli were digital-to-analog converted using a Creative Sound Blaster PCI128 (Creative SB Audigy 2 ZS, Creative Technology Ltd, Singapore) and presented via headphones (HD 265 linear, Sennheiser electronic GmbH & Co. KG, Germany). The level of the noise stimulus was set at 60 dB SPL. Sound intensity was calibrated using a Larson Davis Audiometer Calibration and Electroacoustic Testing System (AUDit and System 824, Larson Davis, Depew, NY, USA). The interaural correlation of the noise stimuli was set to 1. The right-ear noise always started simultaneously with or led the left-ear noise, and different noise samples were used for each trial.

#### Experiment 2

The apparatus and stimuli used in Experiment 2 were similar to those of Experiment 1, except that the interaural correlation of the noise stimuli was set to 1, 0.85, 0.7, 0.55, or 0.4 and the interaural delay was fixed at 0 ms.

The interaural correlations were manipulated via the previously established Asymmetric-Two-Generator method [29,30]. Specifically, two independent noises were constructed by drawing two sets of Gaussian distributed values (sampling frequency = 48 kHz), which was different from that of the noises used in Experiment 1), denoted n_1_(t) and n_2_(t). The noise waveforms presented to the left and right ears [n_L_(t) and n_R_(t), respectively] were constructed by mixing n_1_(t) and n_2_(t) using the following equations:

$$n_{L}(t)=n_{1}(t)$$ Eq 1

and

$$n_{R}(t)=\rho n_{1}(t)+\sqrt{1-\rho^{2}}n_{2}(t)$$ Eq 2

where ρ was 1.0, 0.85, 0.70, 0.55 or 0.40. Note that the actual value of the interaural correlation coefficient may differ slightly from the intended value. The RMS difference between the actual and intended coefficients under each of the conditions was less than 0.01.

### Procedures

#### Experiment 1

The duration threshold with the marker correlation held at 1 was estimated for each of the 5 interaural delays (0, 2, 4, 6, 8 ms), using an adaptive two-interval, two-alternative, forced-choice (2AFC) procedure. Based on our previous studies, the largest interaural delay (8 ms) was set at slightly less than the longest interaural delay (~10 ms) when the binaural gap can be detected [24]. In one interval, the same 1000-ms noise was presented to each ear. In the other interval, the same noise was also presented to each ear except that an independent noise segment was substituted at the temporal middle of the left-ear noise (this substituted noise segment induced the binaural gap). Note that the listeners could not detect any changes during the noise when the noise was delivered monaurally. In each trial, the binaural gap was randomly assigned to one of the two intervals. The participant’s task was to identify which of the two intervals contained the binaural gap by pressing the left-button or right-button on a response box. The gap duration was manipulated using a three-down-one-up procedure [31]. The duration was decreased after three consecutive correct identifications of the interval containing the binaural gap and increased after one incorrect identification. The initial step-size for changing the binaural gap duration was 16 ms, and the step-size was altered by a factor of 0.5 with each reversal of direction until the minimum step-size of 1 millisecond was reached. Feedback was given after each trial via a LCD monitor placed in front of the participant. A run was terminated after ten reversals, and the duration threshold for the session was defined as the arithmetic mean binaural gap duration at the last 6 reversals. For each test condition and participant, the arithmetic mean of the duration thresholds for three runs was calculated as the participant’s duration threshold.

#### Experiment 2

In Experiment 2, the duration thresholds for detecting the binaural gap in the temporal middle of noises with different interaural correlations (1, 0.85, 0.7, 0.55, or 0.4) was tracked using the identical procedures used in Experiment 1. The lowest interaural correlation (0.4) was set at just higher than the smallest interaural correlation (0.3) at which the listeners were able to discriminate it from a reference noise (interaural correlation of 0) [21].

## Results

### Experiment 1

**Fig 1:**
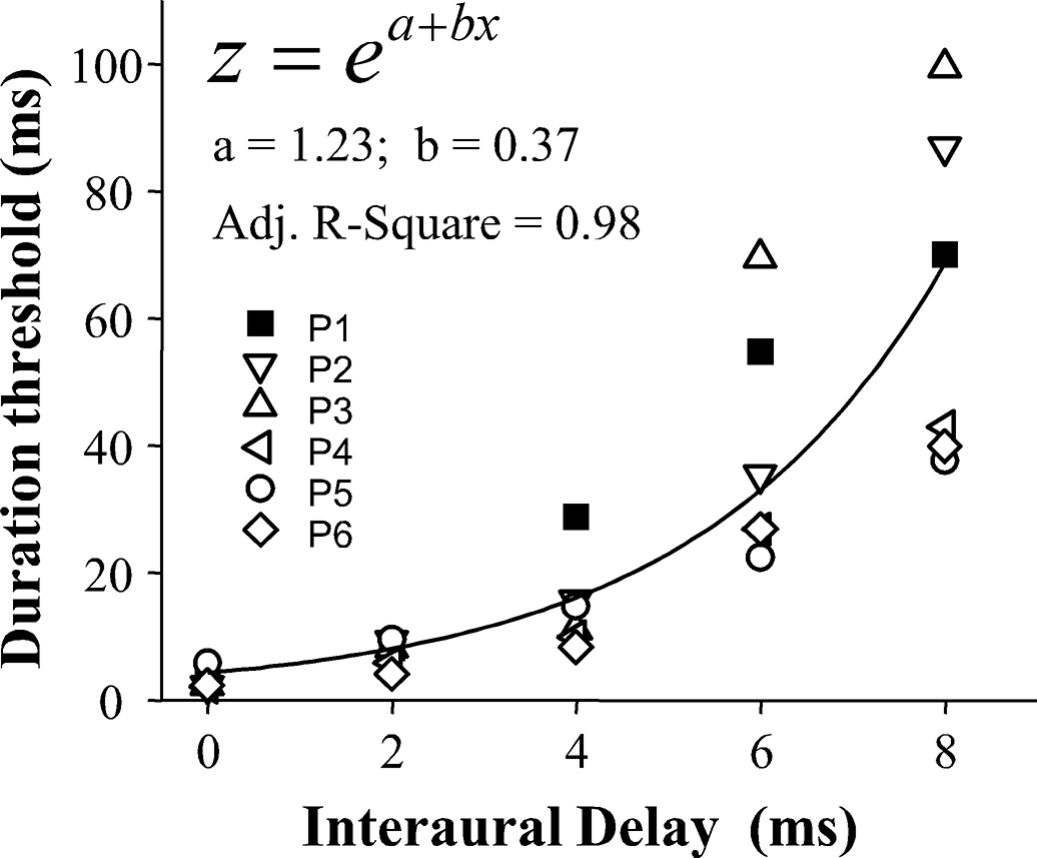
The duration threshold for detecting the binaural gap at five interaural delays (marker interaural correlation = 1). Different symbols represent the duration threshold of each participant. The solid curve shows the best-fitting function for the group-mean duration threshold as a function of the interaural delay. The equation of the best-fitting function is presented in the top left.

All participants succeeded in detecting the binaural gap for each of the 5 interaural delays. Fig 1 shows the duration thresholds for individual participants and the best-fitting function (curve) of the group-mean duration threshold as a function of the interaural delay. As the interaural delay increased, not only the duration threshold increased monotonically at an accelerated rate for each participant, but also the inter-participant variability became larger.

The best-fitting function exhibited the form:

$$z=e^{a+bx}$$ Eq 3

where z is the duration threshold for the interaural delay x; a determines the range of z; b is the coefficient determining the rate of change of the function; e is Euler's constant (2.71828). The values of the parameters a and b are indicated in Fig 1.

A one-way ANOVA illustrates that the effect of interaural delay on the duration threshold was significant (F_4,20_ = 22.297, p < 0.001). Least Significant Difference (LSD) post hoc analysis shows that the duration threshold increased significantly for each step in interaural delay from 0 to 8 ms (all p < 0.05).

### Experiment 2

**Fig 2:**
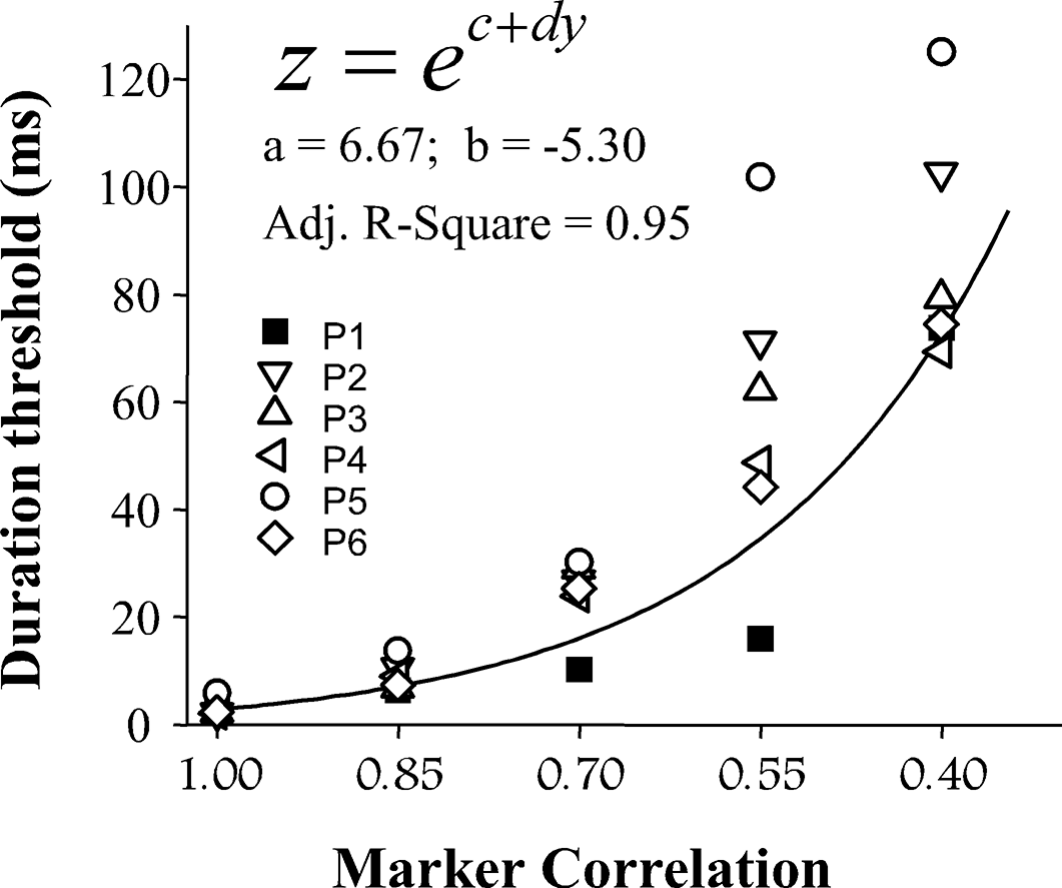
The duration threshold for detecting the binaural gap at five marker correlations (interaural delay = 0 ms). Different symbols represent the duration thresholds of each participant. The solid curve shows the best-fitting function for the group-mean duration threshold as a function of marker correlation. The equation of the best-fitting function is presented in the top left.

All participants were able to detect the binaural gap for each of the marker correlations. Fig 2 shows duration thresholds for individual participants and the best-fitting function (curve) of the group-mean duration threshold as a function of marker correlation. With decreasing correlation of the noise marker, both the duration threshold for each participant and the inter-participant variability increased markedly.

The best-fitting function for Experiment 2 was established in a form as Eq 3:

$$z=e^{c+dy}$$ Eq 4

where z is the duration threshold for the marker correlation y; c determines the range of z; d is the coefficient determining the rate of change of the function; e is Euler's constant (2.71828). The values of the parameters c and d are indicated in the Fig 2.

A one-way ANOVA shows that the effect of marker correlation on the duration threshold was significant (F_4,20_ = 42.903, p < 0.001). LSD post hoc analysis confirms that the duration threshold increased significantly for each step in marker correlation from 1 to 0.4 (all p < 0.05).

## Discussion

The results of Experiment 1 confirmed that the duration threshold increased in exponential fashion as the interaural delay was increased from 0 to 8 ms as found in our previous work [24]. The results of Experiment 2 showed that the duration threshold increased in exponential fashion as the marker correlation decreased from 1 to 0.4. An increased duration threshold implies an increased difficulty to detect the contrast between the dynamic break in interaural correlation and the marker flanking the break.

In this study, the change in duration threshold between the interaural correlation of 0.55 and 0.4 was much larger than that between the interaural correlation of 1.0 and 0.85. This pattern appears to be different from that of the just-noticeable difference (JND) as a function of the reference interaural correlation where there is a large change near the reference interaural correlation of 1.0 and only a small change near the reference interaural correlation of 0.5 [32]. It should be noted that some features of this study were different from those of the previous study [32]. First, in this study since participants needed to detect a dynamic change in interaural correlation, binaural sluggishness [9,20,33,34] would be more effective in affecting the detection. Thus, there was a temporal build up for the process of detecting the correlation change. In addition, in this study the contrast was always between a noise fragment with zero interaural correlation (i.e., the binaural gap) and the noise marker with a non-zero interaural correlation. Some unexpected high sensitivity near interaural correlation of 0.5 was also reported by Rakerd and Hartmann in noise localization [35]. Thus, different binaural tasks may be affected differently by the interaural correlation. Particularly, in future investigation, it is of interest of know how the binaural sluggishness affects the sensitivity to the dynamic contrast in interaural correlation.

The results of both Experiment 1 and Experiment 2 also showed that as either the interaural delay increased or the interaural correlation of the noise marker decreased, the inter-listener variability in duration threshold became larger. These results are in agreement with previous reports that even in younger adults with normal hearing, the inter-listener variability in detecting the binaural gap becomes larger with an increase of the interaural delay [22], particularly when the interaural delay is around the threshold [24,26].

**Fig 3:**
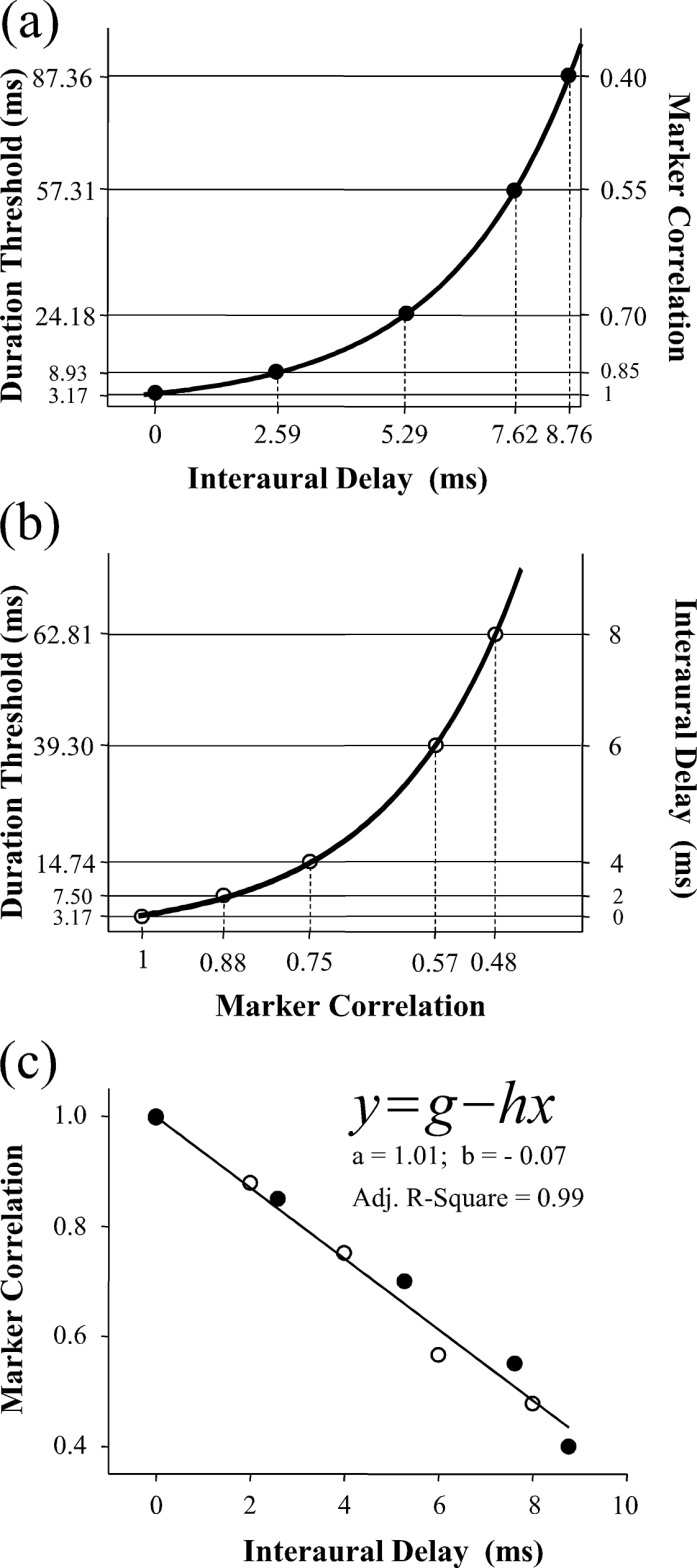
Relationship between the interaural delay and marker correlation on detecting the binaural gap. Top panel (a): The solid curve shows the function for the effect of interaural delay on the duration threshold. The horizontal solid lines represent the duration threshold measured at each of the marker correlations (1, 0.85, 0.7, 0.55, and 0.4); the vertical dashed line represents the interaural delay computed from function at these duration thresholds. The filled circles depict the interaural delay and marker correlation with equivalent effect on the duration threshold. Middle panel (b): The solid curve shows the function for the effect of marker correlation on the duration threshold. The horizontal solid lines represent the duration threshold measured at each of the interaural delays (0, 2, 4, 6 and 8 ms); the vertical dashed line represents the marker correlation computed from function at these duration thresholds. The open circles depict the interaural delay and marker correlation with equivalent effect on the duration threshold. Lower panel (c): the solid line shows the best fitting function between the interaural delays and marker correlations from the five samples from the upper panel (filled circles) and five samples from middle panel (open circles); the equation of the best-fitting function is presented in the top left.

The main goal of this study was to describe the mathematical relationship between the interaural delay and marker correlation. Using the best-fitting function curve obtained from Experiment 1 (Fig 1) and the duration-threshold values measured at the 5 marker correlations (1, 0.85, 0.70, 0.55, 0.40) from Experiment 2, the upper panel of Fig 3 (panel a) displays the 5 interaural delay values (along the abscissa) that are related to the same duration thresholds with the 5 marker correlation values (along the ordinate), respectively.

Similarly, using the best-fitting function curve obtained from Experiment 2 (Fig 2) and the duration-threshold value measured at the 5 interaural delays (0, 2, 4, 6, and 8 ms) from Experiment 1, the middle panel of Fig 3 (panel b) displays the 5 marker correlation values (along the abscissa) that are related to the same duration thresholds with the 5 interaural delay values (along the ordinate), respectively. Consequently, based on the associations between the marker correlation and the interaural delay as presented in panels a and b of Fig 3, the parameterization of the linear relationship between the marker correlation and the interaural delay was determined (panel c of Fig 3):

y=1.01−0.07x Eq 5

where y is the marker correlation when the interaural delay is x (0 ≤ y ≤ 1, and *x* ≥ 0). Note that as described above, to take full advantage of the observed data from the two experiments, the linear relationship between interaural delay and correlation is based on the combination of the function obtained from one experiment and the observed data points from the other experiment, but not completely on the functions from the two experiments. Thus, deviations from linearity exhibit in Fig 3C.

Moreover, theoretically the data from Experiment 1 and 2 should share one point that the interaural delay is zero and interaural correlation is 1. However, the value of the equation in Experiment 1 when the interaural delay is zero is slightly different from the value of the equation in Experiment 2 when the interaural correlation is 1. This may be because of the near miss when the two equations are developed.

In addition, the linear relationship between interaural delay and marker correlation can also be directly obtained from Eqs 3 and 4. Based on the parameters given in each of two equations, the following function can be established:

y=1.03−0.07x Eq 6

Note that the parameter values in Eq 6 are only slightly different from those in Eq 5 because of the near miss

Assuming that y_1_ is the marker correlation when the interaural delay is x_1_, y_2_ is the marker correlation when the interaural delay is another value x_2_, Δy is the difference between y_1_ and y_2_, and Δx is the difference between x_1_ and x_2_. The following linear relationship between Δy and Δx is then obtained:

Δy=-0.065Δx Eq 7

This equation shows that an increase of 1 ms in interaural delay is equivalent to a reduction about 0.07 in marker correlation specific to raising the duration threshold.

Our data clearly indicate that the impact of the interaural delay and that of the marker correlation on detecting a temporal change in interaural correlation are similar and highly related, suggesting a shared mechanism between interaural-delay and interaural-correlation processing. Since the increase of interaural delay has also been proven to cause a deterioration of the binaurally perceptual fusion [10], which depends on interaural correlation, and binaural neurons in the central auditory system are sensitive to both interaural delay and interaural correlation [12–15], establishing new theoretic models will be an important issue in this line of studies.

In the classical model of binaural hearing, there are coincidence detectors that integrate the simultaneously arrived neural pulses from the left and right ears [1]. The interaural delay is coded by delays between fibers from the two ears and the interaural correlation is represented by the magnitude of active coincidences [36,37,38]. However, the existing models mainly put emphasis on the physiological range of interaural delay for the sound-wave propagation across the distance between the ears. Since detection of the BIC can occur at interaural delays far beyond the physiological range of interaural delay [22–27, this study], new models must include certain signal processing components at higher-order perceptual levels, including the primitive auditory memory (PAM) [24,26]. According to the PAM theory, when the interaural delay is progressively increased, the PAM of fine-structure signals from the leading ear progressively decay (i.e., the central representation of fine structures of the noise entering the leading ear becomes more and more diminished), leading to a progressive reduction of the interaural correlation of the central representation of the noises from the two ears.

How important is the PAM for actual hearing? In a (simulated) reverberant environment with multiple people speaking, the perceptual integration of direct speech sound waves with their reflections plays an important role in improving speech perception by inducing a perceived spatial separation between target speech and masking speech [24,26,39,40,41]. The PAM is associated with the ability to perceptually integrate the direct wave from the target source with the reflections of the source, and the integrating ability is critical to perceptually segregate the target source from the other uncorrelated (masking) sources [24,26].

## Conclusions

This study measured and compared the effects of interaural delay and interaural correlation in a group of participants. Our work discovered a linear relationship between the changes in interaural delay and interaural correlation required to produce an equivalent decline of sensitivity to the binaural gap: an increment of 1 ms in interaural delay is equivalent to a reduction about 0.07 in interaural correlation. Future studies may help ascertain whether there is an age-related and/or hearing-loss-related change in the relationship between interaural delay and interaural correlation.

## Supporting Information

S1 File Supporting Information files. (ZIP)
