# Supplementary material for: Similar Impacts of the Interaural Delay and Interaural Correlation on Binaural Gap Detection
Source: PLoS One. 2015 Jun 30;10(6):e0126342. doi: 10.1371/journal.pone.0126342 (PMC4488353; doi:10.1371/journal.pone.0126342)
Supplement: S1 File — (ZIP) [file pone.0126342.s001.zip › Fig. 1 fitting description.pdf]

1

NL Fit (Exp2PMod2) (2015/3/17 01:51:54)

Notes

Description	NL Fit
User Name	user
Operation Time	2015/3/17 01:51:54
Model	Exp2PMod2
Equation	y = exp(a+b*x)
Report Status	New Analysis Report

Parameters

		Value	Standard Error
B	a	1.23585	0.08277
	b	0.37302	0.01794

Iterations Performed = 6
Total Iterations in Session = 6
Fit converged - tolerance criterion satisfied.

Statistics

	B
Number of Points	5
Degrees of Freedom	3
Reduced Chi-Sqr	0.45504
Residual Sum of Squares	1.36513
Adj. R-Square	0.97565
Fit Status	Succeeded(100)

Fit Status Code :
100 : Fit converged

Summary

	a		b		Statistics	
	Value	Error	Value	Error	Reduced Chi-Sqr	Adj. R-Square
B	1.23585	0.08277	0.37302	0.01794	0.45504	0.97565

ANOVA

		DF	Sum of Squares	Mean Square	F Value	Prob>F
B	Regression	2	5771.78317	2885.89159	6341.99743	4.364E-6
	Residual	3	1.36513	0.45504		
	Uncorrected Total	5	5773.14831			
	Corrected Total	4	74.76506			
