# Supplementary material for: Similar Impacts of the Interaural Delay and Interaural Correlation on Binaural Gap Detection
Source: PLoS One. 2015 Jun 30;10(6):e0126342. doi: 10.1371/journal.pone.0126342 (PMC4488353; doi:10.1371/journal.pone.0126342)
Supplement: S1 File — (ZIP) [file pone.0126342.s001.zip › Fig. 2 fitting description.pdf]

1 *NL Fit (Exp2PMod2) (2015/3/17 01:59:45)*

Parameters

		Value	Standard Error
B	a	6.67336	0.26342
	b	-5.30039	0.38707

Iterations Performed = 8
Total Iterations in Session = 8
Fit converged - tolerance criterion satisfied.

Statistics

	B
Number of Points	5
Degrees of Freedom	3
Reduced Chi-Sqr	2.1243
Residual Sum of Squares	6.3729
Adj. R-Square	0.94854
Fit Status	Succeeded(100)

Fit Status Code :
100 : Fit converged

Summary

	a		b		Statistics	
	Value	Error	Value	Error	Reduced Chi-Sqr	Adj. R-Square
B	6.67336	0.26342	-5.30039	0.38707	2.1243	0.94854

ANOVA

		DF	Sum of Squares	Mean Square	F Value	Prob>F
B	Regression	2	11583.54004	5791.77002	2726.43541	1.54705E-5
	Residual	3	6.3729	2.1243		
	Uncorrected Total	5	11589.91295			
	Corrected Total	4	165.13846			
