# Supplementary material for: Similar Impacts of the Interaural Delay and Interaural Correlation on Binaural Gap Detection
Source: PLoS One. 2015 Jun 30;10(6):e0126342. doi: 10.1371/journal.pone.0126342 (PMC4488353; doi:10.1371/journal.pone.0126342)
Supplement: S1 File — (ZIP) [file pone.0126342.s001.zip › Fig. 3 fitting description.pdf]

1 *Linear Fit (2015/4/11 01:00:23)*

Parameters

		Value	Standard Error
B	Intercept	1.00927	0.01645
	Slope	-0.06594	0.00306

Statistics

	B
Number of Points	10
Degrees of Freedom	8
Residual Sum of Squares	0.00699
Adj. R-Square	0.98098

Summary

	Intercept		Slope		Statistics
	Value	Error	Value	Error	Adj. R-Square
B	1.00927	0.01645	-0.06594	0.00306	0.98098

ANOVA

		DF	Sum of Squares	Mean Square	F Value	Prob>F
B	Model	1	0.40675	0.40675	465.25526	2.24799E-8
	Error	8	0.00699	8.74253E-4		
	Total	9	0.41374			
